# Clinical Lecture on the Use of Digitalis in Diseases of the Heart

**Published:** 1875-03-15

**Authors:** H. C. Wood


### CLINICAL LECTURE ON THE USE OF DIGITALIS IN DISEASES OF THE HEART.

DELIVERED AT THE PHILADELPHIA HOSPITAL BY H. C. WOOD, JR., M.D.

*From the Canada Medical Record.*

THERE are, gentlemen, primary physiological facts concerning the action of digitalis which I shall to-day lay down somewhat dogmatically as the premises for the discussion of the subject. I do this because time is wanting in which to give you the proofs of these premises, even if they were not out of place in a clinical lecture-room; and I do it the more willingly because those of you who may be inclined to be skeptical can find these proofs in detail in my treatise upon therapeutics.

By experiments upon the lower animals two things have been definitely ascertained: 1, That in the lower animals digitalis is a very powerful stimulant to the inhibitory apparatus of the heart; and 2, That it is also a powerful stimulant to the muscular substance or its contained ganglia.

We know the first, because after the administration of the drug, when the heart has already been affected by it, and is beating slowly, if the inhibitory nerves be cut, the organ springs, as it were, into an intense rapidity of action. The drug stimulates the cardiac muscle, because the amount of work performed by the heart is vastly increased under its influence, even when the viscus is disconnected from the body. We also know as a probable fact that digitalis causes a general vaso-motor spasm, a contraction of the muscular walls of the vessels. This much for the observations made on the lower animals.

When digitalis is administered to man, the first thing we observe is a diminution in the number of heart-beats, and an alteration of the character of the pulse, which becomes full, and hard, and strong. You can recognize by the feel of the blood-wave that both the force of the contraction of the heart and the amount of blood thrown out during the systole are increased. If the drug is given in poisonous doses, the pulse may, it is true, become rapid, and smaller than normal. The meaning of this can be explained by referring again to the animal. We find that here the same phenomena are observed, and that if a very large dose is given the heart may be suddenly arrested in systole from irritation of the cardiac muscle; before this happens, for a time, the tendency to contract is so great that the systole will occur before the complete filling up of the cavities. Two short, imperfect waves are thus produced instead of one long one: this is the double-beat, forming a dicrotic pulse. In man the "dicrotic pulse" of digitalis is classical, and its mechanism is evidently the same as that of the double arterial wave of the lower animals; instead of a long pause and a full dilatation, the first attempt at diastole is interrupted by an abortive systolic contraction. As in animals, probably in these cases also, the apex of the heart scarcely relaxes at all. Again, a person under the influence of digitalis may have a heart beating 50

or 60 per minute when in a recumbent posture, but on sitting up the pulse may suddenly become weak and mount to 100 or 120. The action of digitalis has been carried in such a case to the point at which an excess will throw stimulation into over-stimulation and imperfect contraction. The act of rising brings an extra strain on the heart, and the muscle loses its power of regular action.

Digitalis, then, in man, by its action on the inhibitory apparatus, prolongs the period of diastole, thus giving time for the ventricles to fill up with more blood than usual, and also increases the muscular power of the heart, so that when it contracts a greater volume of blood is thrown with a greater force into the arterial system. Before we begin to apply these principles, remember also that the vascular system under the control of the vaso-motor nerves is probably kept in a state of contraction by the influence of digitalis.

Almost nothing but common sense is needed now to apply these facts to the treatment of heart diseases. If what has been said is true, digitalis ought to be useful when there is a deficiency of heart-power. Remember that it is not a rag that will stop a leak, and do not fall into the common error of expecting the drug to perform impossibilities. It cannot tighten a leaking valve. It cannot open and smooth down a contracted orifice. In other words, in valvular lesions it can only indirectly remedy the defects; and although often you will get the most surprising results from its use, yet in every case of valvular lesion there comes, sooner or later, a stage when digitalis is powerless. It is when the valves are healthy, and the cardiac failure is due simply to the weakness of the muscular walls that digitalis exerts its most wonderful powers. Nothing is more marvelous in clinical medicine than the relief you can sometimes rapidly afford in cases of simple dilatation of the heart.

